# Did the vaccination campaign in Sweden narrow the gap in hospital-registered COVID-19 cases between foreign-born and Swedish-born individuals? A register-based cohort study of adults

**DOI:** 10.1136/bmjopen-2026-118005

**Published:** 2026-10-27

**Authors:** Magnus Helgesson, Martin Tondel, Tobias Nordquist, Magnus Svartengren

**Affiliations:** 1 Public Health and Caring Sciences, Uppsala Universitet, Uppsala, Sweden; 2 Department of Medical Sciences, Occupational and Environmental Medicine, Uppsala Universitet, Uppsala, Sweden; 3 Occupational and Environmental Medicine, Uppsala University, Uppsala, Sweden; 4 Department of Occupational and Environmental Medicine, Uppsala University Hospital, Uppsala, Sweden

**Keywords:** COVID-19, Vaccination, EPIDEMIOLOGIC STUDIES, Refugees

## Abstract

**Abstract:**

**Objectives:**

To examine differences between foreign-born and Swedish-born individuals regarding four interrelated research questions, 2021–2022: (1) risk factors for hospital-registered COVID-19, (2) vaccination rates for COVID-19, (3) the association between vaccination and COVID-19 and (4) 30-day mortality following COVID-19.

**Design:**

A total-population, register-based cohort study of adults in Sweden.

**Participants:**

All foreign-born (n=1 676 516) and Swedish-born (n=6 037 151) individuals aged 20–87 years, residing in Sweden on 31 December 2019.

**Outcomes:**

Hospital-registered cases of COVID-19, vaccine rates of COVID-19 and the influence of vaccination on hospital-registered and 30-day mortality of COVID-19.

**Methods:**

HRs with 95% CIs for estimating differences between foreign-born and Swedish-born individuals regarding hospital-registered COVID-19 and mortality with or without COVID-19 vaccinations were estimated. Effect estimates were adjusted for age, sex, educational level, household size and occupation.

**Results:**

In working age (20–64 years), women had a higher risk of COVID-19 than men, both among foreign-born (adjusted HR: 1.13; 95% CI 1.10 to 1.16) and Swedish-born individuals (adjusted HR: 1.17; 95% CI 1.15 to 1.20). In crude models, increasing age was strongly associated with COVID-19 (HRs: 1.27–2.91); however, it diminished after adjustment (HRs: 1.07–1.48). Furthermore, elementary school only (adjusted HRs: 1.24–1.66), contact-intensive occupations (adjusted HRs: 1.11–1.97) and larger household size among foreign-born individuals (adjusted HR: 1.60; 95% CI 1.53 to 1.68) were associated with a higher risk of COVID-19 before taking vaccination into account. As of 31 December 2021, foreign-born individuals had substantially lower vaccination coverage (64.1%) than Swedish-born individuals (86.9%). After adjustment, vaccinated foreign-born individuals had a similar risk of hospital-registered COVID-19 as that of Swedish-born individuals. The 30-day mortality rate was generally low, with similar rates between foreign-born and Swedish-born individuals.

**Conclusions:**

The difference in severe COVID-19 between foreign-born and Swedish-born individuals diminished after the vaccine rollout. Remaining risks were associated with low educational level, large household size and certain occupations, while 30-day mortality was similar between foreign-born and Swedish-born individuals.

**STRENGTHS AND LIMITATIONS OF THIS STUDY**

- A population-based cohort including all foreign-born (≈1.6 million) and Swedish-born (>6 million) individuals provided analyses with high statistical power.
- High-quality national registers and within-trade comparisons provided accurate risk estimates with high precision.
- 12-month post-primary vaccination follow-up and PCR-confirmed outcomes minimised misclassification of infection.
- Not including undocumented migrants and asylum seekers and non-hospital cases may underestimate infection burden if case ascertainment differs by birthplace.
- Classifying Swedish-born children of foreign-born parents as Swedish-born, restricting the study to working-age adults and lacking prevaccination infection data may limit generalisability.

## Background

 The COVID-19 pandemic has presented unprecedented challenges to public health systems worldwide, leading to a global race to vaccinate populations to prevent hospitalisation and mortality. As vaccination also protects non-vaccinated individuals through herd immunity, it is a critical public health concern to get as many people as possible vaccinated as quickly as possible.^1^ Sweden is committed to equitable social welfare and comprehensive vaccination programmes for children and adults.^2^ Therefore, in an ideal world, the vaccination campaign would reach all inhabitants simultaneously to combat the COVID-19 pandemic.^3^

Significant differences have been reported in both the prevalence of COVID-19 and mortality due to COVID-19 between foreign-born individuals and those born in Sweden.^4^ This phenomenon has also been observed across various European settings, where a foreign-born population appears to be at high risk of severe COVID-19 outcomes.^5^ Furthermore, segregation in the labour market, where foreign-born individuals often hold jobs that involve frequent contact with others, such as taxi and bus drivers and other service workers, may increase the risk of contracting COVID-19.^6^ Moreover, foreign-born individuals, primarily due to social inequalities, tend to live in more densely populated dwellings than Swedish-born individuals, thereby increasing their risk of exposure to contamination both at work and home.^7^ As foreign-born individuals accounted for approximately one-fifth of Sweden’s total population in 2023, this poses a significant challenge to public health.^8^ Compared with many other Western countries, Sweden has had a disproportionately large share of immigrants from outside Europe, who are largely refugees.^8 9^ Foreign-born individuals from outside Europe are often seen as particularly vulnerable, for example, due to lower childhood health status, limited access to healthcare, such as language barriers and a lack of information on preventive measures.^10^ Consequently, COVID-19 may be even more detrimental to them.^9 10^ Based on the discussion above, vaccination against COVID-19 would be particularly beneficial for foreign-born individuals. Some studies, however, report the opposite: foreign-born individuals, to a lesser extent than the host population, receive different forms of preventive healthcare, with vaccines as a crucial measure.^11–14^ One hypothesis generated from our previous study is that vaccination can narrow the gap in incidence and mortality among hospital-registered COVID-19 cases between foreign-born and Swedish-born individuals, compared with the differences observed in 2020.^4^

Therefore, the aims were to study differences between foreign-born and Swedish-born individuals regarding the following interrelated research questions: (1) risk factors and differences between foreign-born and Swedish-born individuals regarding hospital-registered COVID-19, (2) vaccine rates in 2021 for COVID-19 in foreign-born and Swedish-born individuals, (3) the relationship between vaccine status in 2021 and subsequent 1-year hospital-registered COVID-19 in foreign-born and Swedish-born individuals and (4) differences between vaccinated and unvaccinated foreign-born and Swedish-born individuals in mortality within 30 days of a hospital-registered COVID-19. In all the above relationships, the roles of sex, age, educational level, household size and occupation were considered.

## Methods

### Study population

The baseline cohort for this research comprised all individuals aged 20–87 years born between 1932 and 1999, encompassing both all Swedish-born and all foreign-born individuals in this age group residing in Sweden as of 31 December 2019 (closed cohort). Foreign-born individuals are those born outside Sweden, and Swedish-born individuals are those born in Sweden, thereby including children of immigrants born in Sweden. The study included 1 676 516 foreign-born individuals (835 221 women and 841 295 men) and 6 037 151 Swedish-born individuals (3 000 750 women and 3 036 401 men). Statistics Sweden identified this population using a unique identifier linking personal identity numbers. We measured COVID-19 vaccination coverage from the start of Sweden’s vaccination programme in 2021. Hence, the population was somewhat lower in 2021 than it was when the cohort was defined on 31 December 2019 (66 419 individuals died and 35 493 emigrated in 2020).

### COVID-19 vaccination programme in Sweden

During the COVID-19 pandemic, vaccinations were delivered through Sweden’s publicly funded regional healthcare system, including primary healthcare centres, dedicated vaccination clinics, hospitals and residential care settings. Every vaccine shot was provided at no cost to all citizens, with priority given to vulnerable groups such as elderly persons and individuals with known risk factors for having severe COVID-19. From 1 January 2021, reporting of all COVID-19 vaccinations to the National Vaccination Register maintained by the Public Health Agency of Sweden became mandatory under the Act on Registers of National Vaccination Programmes, Swedish Code of Statutes (SFS) 2012:453, as amended by SFS 2020:1194.^15^ The register contains individual-level information, including personal identity number, vaccination date and vaccine type, enabling nationwide monitoring of vaccination uptake and coverage.^3^ Primary vaccination was defined in this study as two doses of a vaccine, regardless of manufacturer (ie, one basic shot and one booster shot), administered within 6 weeks of the basic shot.^16 17^ Because full protective efficacy after COVID-19 vaccination is not achieved immediately, the immune system needs time to develop an adequate response. Among the two most widely used vaccines in Sweden, full protection occurred 7 days after the second dose with the Pfizer-BioNTech vaccine^16^ and 14 days after the second dose with the Moderna mRNA vaccine.^17^ Therefore, the shortest time to full protection, 7 days after administration of the second dose, was chosen as the primary vaccination date.

### Registers and variables

The unique personal identification number enabled data linking from several national registers. The Patient Register, maintained by the National Board of Health and Welfare, included data on hospital treatment, inpatient healthcare and specialised outpatient healthcare, including diagnoses and visit dates.^18^ The diagnosis of COVID-19 in both inpatient and specialised outpatient healthcare was confirmed by tests coded in the International Classification of Diseases, version 10 (ICD-10),^19^ as the emergency code U07.1^20^ and is hereafter referred to as a hospital-registered COVID-19. Because the prevalence of cardiovascular or pulmonary diseases was similar among both foreign-born and Swedish-born individuals, we did not include additional disorders in the analyses. The cause-of-death register, from the same authority as above, included the date of death.^21^ The National Vaccination Register, maintained by the Public Health Agency of Sweden,^3^ provided information on COVID-19 vaccines, including dates for up to four vaccine doses during the study period.^2^ Finally, the Longitudinal Integrated Database for Health Insurance and Labour Market Studies (LISA), provided by Statistics Sweden, provided information on country of birth, year of birth, sex, educational level, household size and occupation.^22^

The highest achieved educational level was measured in three categories: university (≥12 years), high school (10–12 years) and elementary school (≤9 years). Household size was divided into four categories: one, two, three to four and five or more family members, all aged 0 to 87 years and registered at the same address. From the register, we did not retrieve the age for each household member, only household size in 2019. Occupations are registered annually by employers and categorised according to the Swedish Standard Classification of Occupations (SSYK 2012), a national adaptation of the International Standard Classification of Occupations (ISCO-08).^23^ Here, a hierarchical structure with four code levels reflects the nature of the work and the required skill level. Students and unemployed persons do not have an occupational code and will be treated as a ‘missing value’ in the analyses. Based on incidence, certain occupations with an anticipated higher risk of COVID-19 were identified in our previous study, despite having similar educational backgrounds.^4^ These included SSYK codes beginning with 22, corresponding to health professionals (eg, physicians, nurses, care assistants); 23, corresponding to teaching professionals; 51–53, corresponding to service workers (eg, conductors, cooks, servers) and SSYK codes 8321, 8331, 5112, corresponding to personal transport workers, such as taxi and bus drivers.

### Statistical analyses

The analyses were performed in four separate parts:

1. Hospital-registered COVID-19 in 2021–2022 among individuals of working age (20–64 years) having an active occupation coded in registers on 31 December 2019. Estimates were calculated using HRs with 95% CIs from Cox regression analysis with the Breslow approximation, which accounts for tied survival times. In the adjusted model, the analyses were mutually adjusted for age, sex, educational level, household size and occupation. Age was treated as a continuous variable, while all other covariates were treated as categorical variables. Missing values for the variables are presented in table 1. The analyses were additionally stratified by occupation and sex in a separate table to highlight the impact of occupation on differences in hospital-registered COVID-19 between foreign-born and Swedish-born individuals. Additionally, to put the results in a societal context, a crude analysis was performed across the whole adult population (20–87 years), with results presented by age group in a graph.
2. A descriptive analysis calculated the rate of vaccination for COVID-19. Primary vaccination involves two shots of the vaccine, regardless of brand: a basic dose followed by a booster dose administered within 6 weeks of the first dose. The date of vaccination is defined to occur 1 week after the booster dose is administered. Descriptive analyses of the COVID-19 vaccination programme were performed for the full population aged 20–87 years, by vaccine dose and by foreign-born and Swedish-born status.
3. A hospital-registered COVID-19 case from 12 months after primary vaccination in 2021 was used in a separate analysis. The analyses were calculated in the same way as above under point 1, for a population of working-age adults, aged 20–64 years. Additionally, a crude analysis was performed among individuals aged 20–87 years, presented in age groups, with vaccinated and unvaccinated individuals shown separately.
4. Mortality was defined as a registered death within 30 days of a hospital-registered COVID-19, regardless of the cause, following 12 months from primary vaccination and unvaccinated, respectively.

**Table 1:** Characteristics of all foreign-born individuals living in Sweden, aged 20–87 years, on 31 December 2019 (n=1 676 516) and a reference group of all Swedish-born individuals (n=6 037 151)

	Foreign-born	Swedish-born
Variables*	n (%)	n (%)
Sex		
Men	841 295 (50.2)	3 036 401 (50.3)
Women	835 221 (49.8)	3 000 750 (49.7)
Age		
20–24 years	130 890 (7.8)	451 905 (7.5)
25–29 years	166 633 (9.9)	570 134 (9.4)
30–34 years	212 899 (12.7)	500 818 (8.3)
35–39 years	215 344 (12.8)	433 442 (7.2)
40–44 years	184 508 (11.0)	444 949 (7.4)
45–49 years	159 681 (9.5)	505 086 (8.4)
50–54 years	143 612 (8.6)	531 542 (8.8)
55–59 years	127 683 (7.6)	490 230 (8.1)
60–64 years	101 508 (6.1)	462 106 (7.7)
65–69 years	80 018 (4.8)	452 719 (7.5)
70–74 years	65 579 (3.9)	487 054 (8.1)
75–79 years	45 224 (2.7)	376 619 (6.2)
80–84 years	31 837 (1.9)	236 074 (3.9)
85–87 years	11 100 (0.7)	94 473 (1.6)
Missing value	2505 (0.2)	656 (0.0)
Educational level		
University (≥12 years)	623 877 (37.2)	2 352 975 (39.0)
High school (10–12 years)	563 177 (33.6)	2 807 731 (46.5)
Elementary school (≤9 years)	486 957 (29.1)	875 789 (14.5)
Missing value	2505 (0.2)	656 (0.0)
Household size†		
One	640 880 (38.2)	2 306 128 (38.2)
Two	335 858 (20.0)	1 713 380 (28.4)
Three or four	512 767 (30.6)	1 701 588 (28.2)
Five or more	184 506 (11.0)	315 399 (5.2)
Missing value	2437 (0.2)	299 (0.0)
Occupation		
Other occupations‡	543 958 (32.5)	2 490 753 (41.3)
Teaching professionals§	58 528 (3.5)	263 076 (4.4)
Service workers¶	261 540 (15.6)	770 605 (12.8)
Personal transport workers**	22 167 (1.3)	20 561 (0.3)
Health professionals††	35 712 (2.1)	165 032 (2.7)
Not of working age (>64 years)	233 758 (13.9)	1 646 939 (27.3)
Missing value‡‡	518 416 (30.9)	679 886 (11.3)
Total study population	1 676 516 (100)	6 037 151 (100)

* All variables were measured on the 31st of December 2019

† Persons 0 to 87 years of age

‡ All SSYK except 22, 23, 51-53, 8321, 8331, 5112

§ SSYK 23

¶ SSYK 51-53

** SSYK 8321, 8331, 5112

†† SSYK 22

‡‡ Includes e.g. students and unemployed individuals, as they don’t have an active occupational code

All statistical analyses were conducted using the SAS statistical package V.9.4 (SAS).

### Patient and public involvement

None.

## Results

### Characteristics

The proportions of men and women were equal among both foreign-born and Swedish-born individuals (table 1). In general, foreign-born individuals were younger than Swedish-born individuals. Regarding the highest completed educational level, foreign-born individuals had a higher proportion with elementary school as their highest completed education, but a lower proportion who had graduated from high school than Swedish-born individuals. Furthermore, 11% of foreign-born individuals lived in households with five or more persons, compared with 5.2% of Swedish-born individuals (table 1). There was a considerable difference in the proportion of personal transport workers among foreign-born individuals (1.3%) compared with Swedish-born individuals (0.3%). Also, the proportion of service workers was somewhat higher among foreign-born individuals.

### Hospital-registered COVID-19

#### Comparison of hospital-registered COVID-19 cases between foreign and Swedish-born individuals

In the crude model, the risk of hospital-registered COVID-19 in 2021 was substantially higher among foreign-born individuals aged 20–87 years (HRs up to 3) than among Swedish-born individuals (online supplemental figure 1). However, the risk difference of hospital-registered COVID-19 was considerably lower among individuals aged over 75 years. In 2022, the risk difference between foreign-born and Swedish-born individuals levelled out across all age groups, resulting in HRs <1.5 for ages 20 to 87.

When stratifying the analysis by occupational group and sex in a working-age population aged 20–64 years, the risk of hospital-registered COVID-19 was consistently higher among foreign-born individuals across all occupational groups compared with Swedish-born individuals (table 2). For example, foreign-born personal transport workers and teaching professionals of both sexes had an increased risk of being diagnosed with COVID-19 that was twice that of their Swedish counterparts in these occupational groups from 1 January 2021 to 31 December 2022. Furthermore, foreign-born service workers, both men and women, had just over a 60% higher risk of hospital-registered COVID-19 cases than Swedish-born individuals in the same occupations.

**Table 2:** HRs for hospital-registered COVID-19 (U07.1), stratified on occupation and sex, for foreign-born individuals with Swedish-born individuals serving as reference in each cell with 95% CI

			Crude	Adjusted*
Occupation†		Cases‡	HR (95% CI)	HR (95% CI)
Other occupations§	Women	2798/7292	1.81 (1.73 to 1.89)	1.76 (1.68 to 1.84)
	Men	4207/10 903	1.77 (1.71 to 1.84)	1.92 (1.85 to 1.99)
Teaching Professionals¶	Women	562/1671	1.75 (1.59 to 1.93)	1.77 (1.61 to 1.95)
	Men	245/473	1.77 (1.52 to 2.07)	2.00 (1.70 to 2.34)
Service workers**	Women	2898/5679	1.65 (1.58 to 1.72)	1.62 (1.54 to 1.69)
	Men	1164/1710	1.67 (1.55 to 1.80)	1.63 (1.51 to 1.76)
Personal transport workers††	Women	27/49	2.03 (1.26 to 3.25)	2.13 (1.32 to 3.45)
	Men	488/207	1.73 (1.47 to 2.04)	1.98 (1.67 to 2.36)
Health Professionals‡‡	Women	376/1805	1.15 (1.03 to 1.29)	1.17 (1.04 to 1.30)
	Men	215/394	1.56 (1.32 to 1.84)	1.55 (1.31 to 1.83)
Missing value§§	Women	4728/4020	1.38 (1.32 to 1.44)	1.34 (1.28 to 1.40)
	Men	3595/4181	1.30 (1.25 to 1.36)	1.31 (1.25 to 1.37)

* Adjusted for age, educational level, and household size

† Measured on the 31st of December, 2019

‡ Cases Foreign-born/Swedish-born

§ All SSYK except 22, 23, 51-53, 8321, 8331, 5112

¶ SSYK 23

** SSYK 51-53

†† SSYK 8321, 8331, 5112

‡‡ SSYK 22

§§ Includes e.g. students and unemployed individuals, as they don’t have an active occupational code

#### Risk factors for a hospital-registered COVID-19

Among individuals in working age, 20–64 years, the risk of having hospital-registered COVID-19 was slightly higher among foreign-born and Swedish-born women than their male counterparts (table 3). In the crude model, age was a significant predictor of hospital-registered COVID-19. In the adjusted model, however, the effect of age was much attenuated. Having an elementary or high school education as the highest level of education increased the risk of hospital-registered COVID-19 compared with those with a university education. Being 2–5 persons (0–87 years) in a household increased the risk of hospital-registered COVID-19 among foreign-born individuals but not among Swedish-born individuals. Furthermore, after adjustment for covariates, individuals working in contact occupations, such as personal transport workers, healthcare professionals and service workers, still had a higher risk of hospital-registered COVID-19 than those in other occupations (table 3).

**Table 3:** HRs with 95% CI of hospital-registered COVID-19 (U07.1) in working-age (20–64 years) foreign-born individuals and Swedish-born individuals, 1 January 2021–31 December 2022

	Crude model		Adjusted model*	
	**Foreign-born**	**Swedish-born**	**Foreign-born**	**Swedish-born**
**Variables** †	HR (95% CI)	HR (95% CI)	HR (95% CI)	HR (95% CI)
Sex				
Men (ref)				
Women	1.16 (1.13 to 1.20)	1.20 (1.17 to 1.22)	1.13 (1.10 to 1.16)	1.17 (1.15 to 1.20)
Age				
20–24 years (ref)				
25–29 years	1.27 (1.18 to 1.37)	1.34 (1.27 to 1.41)	1.22 (1.11 to 1.33)	1.32 (1.25 to 1.41)
30–34 years	1.32 (1.23 to 1.42)	1.44 (1.37 to 1.51)	1.11 (0.98 to 1.25)	1.33 (1.22 to 1.45)
35–39 years	1.47 (1.37 to 1.58)	1.33 (1.26 to 1.40)	1.08 (0.92 to 1.27)	1.14 (1.01 to 1.28)
40–44 years	1.62 (1.51 to 1.74)	1.39 (1.32 to 1.47)	1.07 (0.87 to 1.31)	1.10 (0.95 to 1.28)
45–49 years	1.90 (1.77 to 2.03)	1.65 (1.57 to 1.73)	1.15 (0.90 to 1.47)	1.19 (0.99 to 1.44)
50–54 years	2.19 (2.04 to 2.35)	1.88 (1.79 to 1.97)	1.25 (0.93 to 1.67)	1.23 (0.99 to 1.53)
55–59 years	2.48 (2.31 to 2.64)	2.05 (1.95 to 2.15)	1.34 (0.95 to 1.88)	1.20 (0.93 to 1.55)
60–64 years	2.91 (2.71 to 3.12)	2.15 (2.05 to 2.25)	1.48 (1.01 to 2.18)	1.12 (0.84 to 1.49)
Educational level				
University (≥12 years) (ref)				
High school (10 to 12 years)	1.25 (1.21 to 1.29)	1.20 (1.17 to 1.23)	1.21 (1.17 to 1.26)	1.27 (1.23 to 1.30)
Elementary school (≤9 years)	1.21 (1.17 to 1.25)	1.71 (1.64 to 1.76)	1.24 (1.19 to 1.28)	1.66 (1.60 to 1.72)
Household size‡				
One (ref)				
Two	1.44 (1.38 to 1.50)	1.11 (1.08 to 1.14)	1.16 (1.12 to 1.22)	0.93 (0.90 to 0.95)
Three or four	1.46 (1.41 to 1.51)	0.95 (0.93 to 0.97)	1.45 (1.40 to 1.50)	1.00 (0.98 to 1.03)
Five or more	1.62 (1.55 to 1.69)	0.96 (0.92 to 1.00)	1.60 (1.53 to 1.68)	1.05 (1.01 to 1.10)
Occupation				
Other occupations (ref)§				
Teaching professionals¶	1.08 (1.00 to 1.16)	1.11 (1.06 to 1.16)	1.11 (1.03 to 1.20)	1.18 (1.13 to 1.24)
Service workers**	1.20 (1.15 to 1.25)	1.31 (1.28 to 1.35)	1.13 (1.09 to 1.18)	1.25 (1.21 to 1.29)
Personal transport workers††	1.79 (1.64 to 1.96)	1.71 (1.51 to 1.93)	1.54 (1.41 to 1.69)	1.46 (1.29 to 1.66)
Health professionals‡‡	1.29 (1.18 to 1.40)	1.83 (1.75 to 1.91)	1.28 (1.17 to 1.40)	1.97 (1.88 to 2.07)
Missing value§§	1.27 (1.23 to 1.31)	1.67 (1.62 to 1.72)	1.26 (1.22 to 1.30)	1.60 (1.55 to 1.64)

* Mutually adjusted for all variables in the table, i.e., age, sex, educational level, household size, and occupation

† All variables were measured on the 31st of December 2019

‡ Persons 0 to 87 years of age

§ All SSYK except 22, 23, 51-53, 8321, 8331, 5112

¶ SSYK 23

** SSYK 51-53

†† SSYK 8321, 8331, 5112

‡‡ SSYK 22

§§ Includes e.g. students and unemployed individuals, as they don’t have an active occupational code

### Primary vaccination rates for COVID-19

Among foreign-born individuals, 64.1% had primary vaccine coverage as of 31 December 2021, compared with 86.9% among Swedish-born individuals at the same time (figure 1). When broken down by age group (20–64 years), the proportion of unvaccinated foreign-born men in 2021–2022 was up to three times that of their Swedish-born counterparts (table 4). The gap between foreign-born and Swedish-born women, however, was not as large as among men. The most significant difference was observed among individuals aged 20–29 years (83% among Swedish-born individuals vs 57% among foreign-born individuals). Within contact occupations, foreign-born personal transport workers and service workers had about twice the proportion of unvaccinated individuals, foreign-born health professionals about three times and foreign-born teaching professionals about four times the proportion of unvaccinated individuals compared with their Swedish-born counterparts.

**Figure 1:**
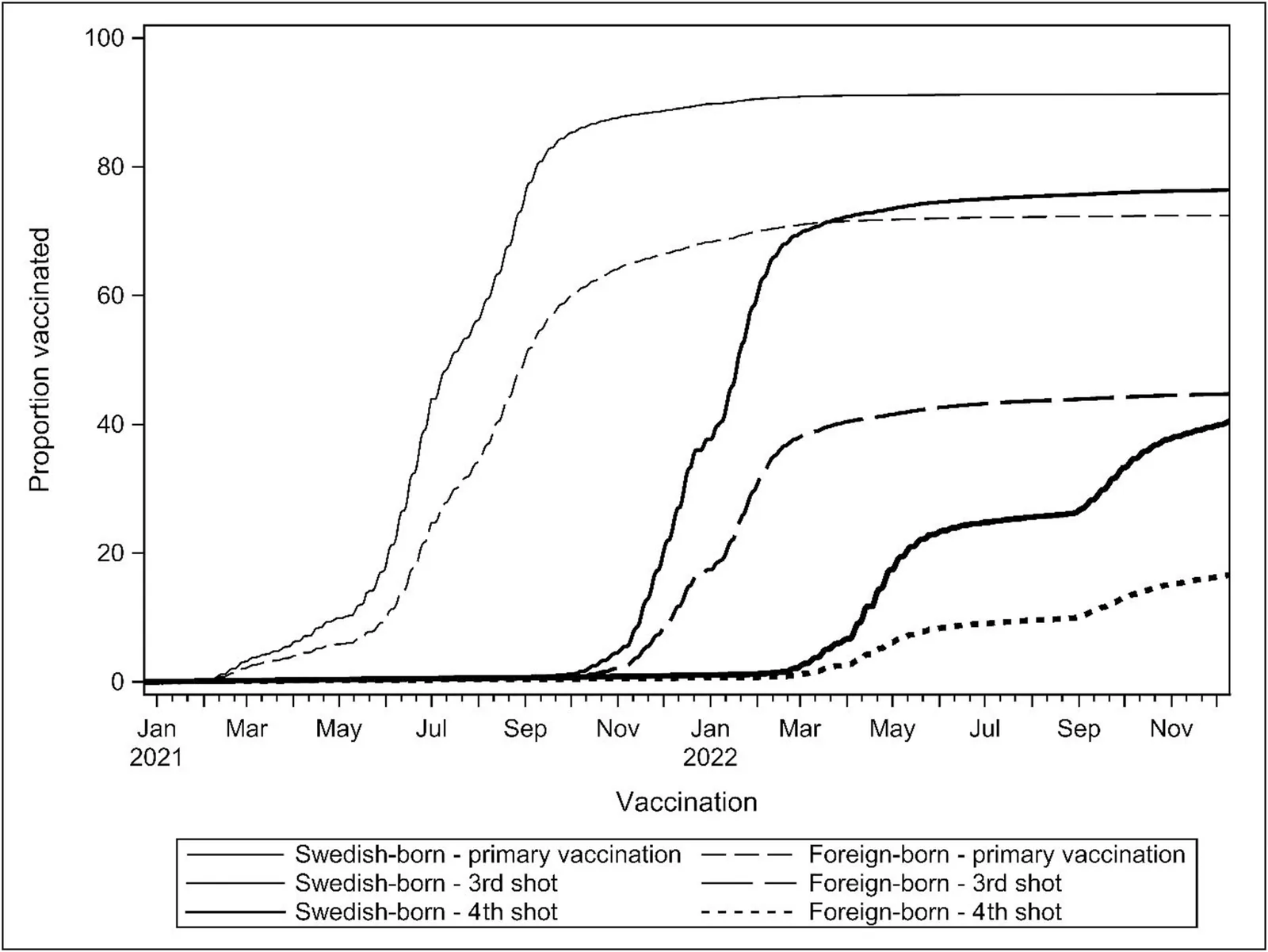
The timeline for vaccination with the first two doses (primary vaccination) and the third and fourth doses against COVID-19 from 1 January 2021 to 31 December 2022.

**Table 4:** Number and proportion of vaccinations across categories from 1 January 2021 to 31 December 2022, for foreign-born individuals of working age (20–64 years) compared with Swedish-born individuals in the same age group

	Primary vaccination*		No vaccination†	
	**Foreign-born**	**Swedish-born**	**Foreign-born**	**Swedish-born**
**Variables‡**	**n (%)**	**n (%)**	**n (%)**	**n (%)**
Sex				
Men	482 148 (65.6)	1 969 319 (87.7)	253 037 (34.4)	276 406 (12.3)
Women	507 188 (71.7)	1 928 662 (89.9)	200 349 (28.3)	215 662 (10.1)
Age				
20–24 years	75 079 (57.4)	374 138 (82.8)	55 810 (42.6)	77 762 (17.2)
25–29 years	95 671 (57.4)	471 057 (82.6)	70 960 (42.6)	99 069 (17.4)
30–34 years	132 547 (62.3)	425 383 (84.9)	80 351 (37.7)	75 429 (15.1)
35–39 years	143 719 (66.7)	381 638 (88.1)	71 620 (33.3)	51 792 (11.9)
40–44 years	129 707 (70.3)	401 712 (90.3)	54 798 (29.7)	43 222 (9.7)
45–49 years	118 421 (74.2)	464 786 (92.0)	41 257 (25.8)	40 288 (8.0)
50–54 years	110 592 (77.0)	490 925 (92.4)	33 017 (20.7)	40 605 (7.1)
55–59 years	101 275 (79.3)	455 154 (92.9)	26 399 (18.9)	35 038 (6.2)
60–64 years	82 325 (81.1)	433 188 (93.8)	19 174 (18.9)	28 863 (6.2)
Educational level				
University (≥12 years)	414 222 (73.7)	1 754 897 (93.4)	147 833 (26.3)	124 070 (6.6)
High school (10–12 years)	325 320 (69.3)	1 829 275 (86.8)	144 395 (30.7)	278 307 (13.2)
Elementary school (≤9 years)	248 687 (60.9)	313 719 (77.8)	159 855 (39.1)	89 640 (22.2)
Household size§				
One	316 159 (60.0)	1 379 026 (86.7)	210 707 (40.0)	211 908 (13.3)
Two	172 965 (74.6)	756 248 (90.5)	58 765 (25.4)	79 635 (9.5)
Three or four	367 889 (73.8)	1 487 775 (90.3)	130 292 (26.2)	160 709 (9.7)
Five or more	131 216 (71.5)	274 842 (87.4)	52 319 (28.5)	39 765 (12.6)
Occupation				
Other occupations¶	394 857 (72.6)	2 264 086 (90.9)	149 101 (27.4)	226 664 (9.1)
Teaching professionals**	46 287 (79.1)	248 454 (94.4)	12 241 (20.9)	14 622 (5.6)
Service workers††	196 668 (75.2)	673 560 (87.4)	64 871 (24.8)	97 045 (12.6)
Personal transport workers‡‡	16 759 (75.6)	17 657 (86.9)	5408 (24.4)	2904 (14.1)
Health professionals§§	31 071 (87.0)	159 025 (96.4)	4641 (13.0)	6007 (3.6)
Missing value¶¶	302 587 (58.4)	535 109 (78.7)	215 821 (41.6)	144 775 (21.3)

* Two shots of vaccine during 2021

† Up to one shot (mostly none) of the vaccine during 2021

‡ All variables were measured on the 31st of December, 2019

§ Persons 0 to 87 years of age

¶ All SSYK except 22, 23, 51-53, 8321, 8331, 5112

** SSYK 23

†† SSYK 51-53

‡‡ SSYK 8321, 8331, 5112

§§ SSYK 22

¶¶ Includes also students and unemployed individuals, without an active occupational code

### The relationship between vaccine status and hospital-registered COVID-19

In the crude model, the risk of hospital-registered COVID-19 among vaccinated individuals from 12 months after primary COVID-19 vaccination in 2021 was significantly higher among foreign-born individuals than among Swedish-born individuals, in both sexes and across most age categories (online supplemental figure 2). The most significant difference between foreign-born and Swedish-born individuals was observed among those in the upper working-age group (55–64 years). However, after full adjustment, only some slight differences in effect remained between vaccinated foreign-born and Swedish-born individuals of working age, mainly in upper working age (table 5). Foreign-born teaching professionals, service workers, personal transport workers, and individuals in other occupations still had a statistically significantly higher risk compared with Swedish-born individuals in the same occupational groups.

**Table 5:** HRs for hospital-registered COVID-19 (U07.1) for foreign-born individuals with Swedish-born individuals serving as reference in each cell with 95% CI

	Primary vaccination in 2021*		No vaccination in 2021†	
	**Crude**	**Adjusted‡**	**Crude**	**Adjusted‡**
Variables§	HR (95% CI)	HR (95% CI)	HR (95% CI)	HR (95% CI)
Sex				
Men	1.37 (1.29 to 1.46)	1.19 (1.12 to 1.27)	1.25 (1.21 to 1.30)	1.22 (1.17 to 1.27)
Women	1.25 (1.19 to 1.31)	1.13 (1.07 to 1.19)	1.29 (1.24 to 1.34)	1.27 (1.22 to 1.31)
Age				
20–24 years	1.26 (1.08 to 1.48)	1.17 (0.99 to 1.39)	1.20 (1.09 to 1.31)	1.20 (1.09 to 1.33)
25–29 years	1.15 (1.02 to 1.30)	1.01 (0.88 to 1.14)	1.15 (1.07 to 1.24)	1.20 (1.11 to 1.29)
30–34 years	1.05 (0.95 to 1.17)	0.98 (0.87 to 1.09)	1.22 (1.13 to 1.32)	1.24 (1.15 to 1.34)
35–39 years	1.25 (1.13 to 1.39)	1.16 (1.04 to 1.30)	1.36 (1.25 to 1.48)	1.35 (1.23 to 1.47)
40–44 years	1.22 (1.08 to 1.37)	1.04 (0.92 to 1.18)	1.30 (1.19 to 1.41)	1.27 (1.16 to 1.38)
45–49 years	1.35 (1.21 to 1.52)	1.11 (0.99 to 1.26)	1.32 (1.22 to 1.44)	1.28 (1.18 to 1.40)
50–54 years	1.40 (1.25 to 1.56)	1.17 (1.04 to 1.32)	1.33 (1.23 to 1.44)	1.26 (1.16 to 1.37)
55–59 years	1.49 (1.34 to 1.66)	1.24 (1.11 to 1.39)	1.41 (1.30 to 1.53)	1.32 (1.21 to 1.44)
60–64 years	1.70 (1.53 to 1.89)	1.42 (1.27 to 1.59)	1.36 (1.25 to 1.48)	1.30 (1.19 to 1.42)
Educational level				
University (≥12 years)	1.24 (1.17 to 1.32)	1.22 (1.14 to 1.29)	1.17 (1.11 to 1.23)	1.23 (1.16 to 1.29)
High school (10 to 12 years)	1.37 (1.28 to 1.46)	1.22 (1.14 to 1.30)	1.53 (1.47 to 1.60)	1.41 (1.36 to 1.47)
Elementary school (≤9 years)	1.03 (0.95 to 1.12)	0.95 (0.87 to 1.04)	1.04 (0.99 to 1.10)	1.01 (0.96 to 1.07)
Household size¶				
One	1.13 (1.05 to 1.21)	0.97 (0.90 to 1.04)	0.88 (0.84 to 0.92)	0.96 (0.92 to 1.01)
Two	1.57 (1.45 to 1.71)	1.39 (1.27 to 1.52)	1.26 (1.18 to 1.34)	1.20 (1.12 to 1.28)
Three or four	1.36 (1.28 to 1.45)	1.21 (1.13 to 1.29)	1.68 (1.60 to 1.76)	1.52 (1.45 to 1.59)
Five or more	1.28 (1.14 to 1.44)	1.10 (0.96 to 1.27)	1.87 (1.73 to 2.04)	1.60 (1.47 to 1.75)
Occupation				
Other occupations**	1.26 (1.18 to 1.34)	1.22 (1.14 to 1.31)	1.35 (1.29 to 1.41)	1.34 (1.28 to 1.40)
Teaching professionals††	1.45 (1.21 to 1.72)	1.42 (1.18 to 1.70)	1.11 (0.96 to 1.28)	1.23 (1.06 to 1.43)
Service workers‡‡	1.15 (1.06 to 1.25)	1.17 (1.07 to 1.27)	1.50 (1.42 to 1.59)	1.45 (1.36 to 1.54)
Personal transport workers§§	1.17 (0.80 to 1.70)	1.33 (0.88 to 2.01)	1.45 (1.15 to 1.84)	1.48 (1.15 to 1.91)
Health professionals¶¶	0.94 (0.80 to 1.10)	0.97 (0.82 to 1.13)	1.18 (0.97 to 1.43)	1.18 (0.97 to 1.42)
Missing value***	1.14 (1.07 to 1.22)	1.08 (1.00 to 1.16)	1.08 (1.03 to 1.13)	1.07 (1.02 to 1.12)

* Two shots of vaccine from January 1 to December 31, 2021

† Up to one shot (mostly none) of vaccine from January 1 to December 31, 2021

‡ Mutually adjusted for all variables in the table, i.e., age, sex, educational level, household size, and occupation

§ All variables were measured on December 31, 2019

¶ Persons 0 to 87 years

** All SSYK except 22, 23, 51-53, 8321, 8331, 5112

†† SSYK 23

‡‡ SSYK 51-53

§§ SSYK 8321, 8331, 5112

¶¶ SSYK 22

*** Includes also students and unemployed individuals, without an active occupational code

When comparing individuals who did not receive a COVID-19 vaccination in 2021, a slightly higher risk of hospital-registered COVID-19 was observed in the crude model among foreign-born individuals, particularly among those of working age (online supplemental figure 2). Compared with those taking primary vaccination, the HRs for unvaccinated foreign-born individuals were similarly higher for hospital-registered COVID-19 among working-age individuals, but lower among those aged 65 and older (online supplemental figure 3). After full adjustment in the unvaccinated group, foreign-born individuals generally still had a higher risk of hospital-registered COVID-19 than Swedish-born individuals (table 5). Statistically significant differences in adjusted HR were observed across occupational groups, indicating that the risk of hospital-registered COVID-19 was higher among foreign-born than among Swedish-born individuals within the same occupation.

### Mortality within 30 days of a hospital-registered COVID-19

In general, mortality within 30 days after a hospital-registered COVID-19 was relatively low in this working-age population, with just over 1000 cases combined across 2021 and 2022 (table 6). Mortality, both among those with primary vaccination and those who were unvaccinated, was similar or lower after adjustment, among foreign-born individuals compared with Swedish-born individuals.

**Table 6:** HRs for mortality for foreign-born individuals with Swedish-born individuals serving as reference in each cell with 95% CI

	Primary vaccination in 2021*			No vaccination in 2021†		
		Crude	Adjusted‡		Crude	Adjusted‡
	Cases§	HR (95% CI)	HR (95% CI)	Cases§	HR (95% CI)	HR (95% CI)
**All**	37/125	1.20 (0.83 to 1.74)	0.82 (0.56 to 1.21)	366/487	0.85 (0.74 to 0.97)	0.86 (0.75 to 1.00)
Women	17/54	1.25 (0.72 to 2.15)	0.69 (0.38 to 1.24)	105/155	0.75 (0.59 to 0.97)	0.63 (0.49 to 0.82)
Men	20/71	1.17 (0.71 to 1.93)	0.92 (0.54 to 1.55)	261/332	0.90 (0.77 to 1.06)	0.98 (0.82 to 1.16)

* Two shots of vaccine from January 1 to December 31, 2021

† Up to one shot (mostly none) of vaccine from January 1 to December 31, 2021

‡ Adjusted for age, educational level, household size, and occupation

§ Cases Foreign-born/Swedish-born

## Discussion

### Main findings

Foreign-born individuals consistently had a higher risk of hospital-registered COVID-19 in 2021 and 2022 combined, although the risk differences were smaller in 2022 than in 2021. Many of these associations, however, were explained by factors such as older age (though not in the working-age population, aged 20–64 years), female sex, lower educational attainment, larger household size and contact-intensive occupations. Foreign-born individuals had a substantially lower primary vaccination rate than Swedish-born individuals. Differences in vaccination also accounted for some of the differences in hospital-registered COVID-19 cases between foreign-born and Swedish-born individuals. However, adjustment for sociodemographic/socioeconomic factors eliminated much of those differences between vaccinated foreign-born and Swedish-born individuals. Among the unvaccinated, foreign-born individuals, however, had higher rates of hospital-registered COVID-19. In general, the 30-day mortality rate was relatively low during 2021 and 2022. Despite a higher rate of hospital-registered COVID-19 cases among foreign-born individuals than among Swedish-born individuals, foreign-born individuals experienced similar or lower mortality rates in 2021–2022 than Swedish-born individuals, regardless of vaccination status.

### Hospital-registered COVID-19

Higher age appears to be one of the most decisive factors in COVID-19, as severe infections are most common among individuals aged 65 and older. This fact was established early in the pandemic, which is also why mortality is relatively low in the working-age population.^24 25^ Age was also not a decisive factor among working-age individuals, where other factors seemed more important. Recommendations from authorities on preventing the spread of infection in the community, primarily targeting older individuals and those with pre-existing conditions, may have reduced the risk of COVID-19 among an ageing population during the pandemic.^25^ Our data show that the differences between foreign-born and Swedish-born individuals are much lower among individuals aged 70 years or older in 2021.

Women appear to have a slightly higher risk of hospital-registered COVID-19 than men, both in the foreign-born and Swedish-born population. Several studies suggest that gendered exposure patterns, occupational roles and social determinants may contribute to this difference.^26 27^ For example, women are overrepresented in frontline and caregiving professions, which increases their likelihood of encountering the virus during peak transmission periods.^27^ Additionally, a study from Scotland shows that women in specific job sectors experience elevated hospital admission risks compared with men, highlighting the role of workplace conditions in shaping vulnerability.^26^ These findings underscore the importance of considering gender differences in exposure when interpreting COVID-19 risk patterns and designing equitable public health responses.

Household size was an important factor for hospital-registered COVID-19 cases among foreign-born individuals. We found that foreign-born individuals, to a greater extent, live in households with five or more individuals. Social inequality and barriers to homeownership may significantly contribute to the high proportion of foreign-born individuals living in crowded households.^7^ A study found that foreign-born individuals are also more likely to live in substandard housing, with mould and moisture present in 50% of foreign-born households in Malmö, a city in southern Sweden.^28^ Indoor smoking was highly prevalent among foreign-born individuals, and the risk of having atopic disorders like asthma was increased among them.^28^ This, together, may increase the risk of transmission of the infection when multiple generations live in the same household.

Foreign-born teaching professionals, personal transport workers and service workers were especially vulnerable to hospital-registered COVID-19. This indicates that foreign-born individuals in those occupations might work in environments with higher levels of virus, or they may be more prone to illness than Swedish-born individuals. During the pandemic, for example, personal transport workers were exposed to a high risk of infection while sometimes transporting ill people and individuals with symptom-free COVID-19. The reason for this increased risk of contagion can be multifaceted.

### Primary vaccination rates for COVID-19

Foreign-born individuals had a substantially lower primary vaccination rate than Swedish-born individuals. The difference in COVID-19 vaccination rates between foreign-born and Swedish-born people was most pronounced among working-age people. The difference was also high among workers in contact occupations, such as personal transport workers, service workers and teaching professionals, who face a high risk of contracting the coronavirus. Moreover, foreign-born individuals seem to use healthcare differently than host populations in European countries.^13^ They tend to have lower attendance and referral rates for preventive measures, such as vaccination, mammography and cervical cancer screening, but equal or higher attendance rates for visits to general practitioners or specialised healthcare.^12–14^ Differences in general vaccination rates between foreign- and Swedish-born individuals have been reported.^14^ For example, a lower vaccination rate among foreign-born individuals is observed for human papillomavirus vaccination.^11^ Furthermore, a Norwegian study concluded that lower trust in authorities and healthcare explained Norway’s lower vaccination rate among foreign-born individuals.^12^ Moreover, the outreach of efforts and information was a decisive factor in the success of vaccination among foreign-born individuals in Norway.^12^

### The relationship between vaccine status and hospital-registered COVID-19

The crude analyses suggest that COVID-19 vaccination did not fully attenuate the elevated risk observed among foreign-born individuals compared with those born in Sweden. This initial finding could be interpreted as indicating the limited effectiveness of the vaccination programme in reducing disparities between these groups. However, once models were adjusted for sociodemographic/socioeconomic factors, such as age, sex, educational level, household size and occupation, the excess risk associated with being foreign-born was mostly eliminated. These results indicate that underlying differences in population composition may significantly affect the vaccine campaign’s impact. The attenuation of the association after adjustment underscores the importance of accounting for social determinants of health when evaluating disparities in infectious disease outcomes. It also suggests that targeted public health strategies that address structural and socioeconomic inequalities may be essential to reducing the disproportionate burden of COVID-19 among foreign-born populations.^29^ Vaccine hesitance has been reported to be high within some groups of foreign-born individuals.^12^ Among health professionals, where vaccination rates were high regardless of origin, the risk of hospital-registered COVID-19 was more similar between foreign-born and Swedish-born individuals than in other contact occupations. The differences in vaccination rate might hence be decisive for the differences in COVID-19 cases between foreign-born and Swedish-born individuals.

In 2022, there was just a slight difference between foreign-born and Swedish-born individuals in hospital-registered COVID-19 cases. The risk difference between foreign-born and Swedish-born individuals was therefore much lower than that shown in our previous study for the first year of the pandemic, 2020.^4^ In the unvaccinated group, confounding by other factors appears less pronounced than in the vaccinated group, suggesting a more similar demographic profile between foreign-born and Swedish-born individuals.

### Mortality within 30 days of a hospital-registered COVID-19

Foreign-born individuals tended to have a similar or lower rate of mortality within 30 days after getting a diagnosis of COVID-19 in 2021 and 2022 compared with Swedish-born individuals, regardless of vaccination. Still, foreign-born individuals had a slightly higher probability of hospital-registered COVID-19 cases. Numerous studies have observed this paradox, and various explanations have been proposed to account for it, including health selection, cultural factors, dietary habits or flaws in data collection.^30–32^ The opposite, however, was found in a previous study conducted by our research group in the first year of the pandemic.^4^ Foreign-born individuals had, in 2020, up to five times higher risk of mortality 30 days after being diagnosed with COVID-19 compared with Swedish-born individuals.^4^ This study also notes that the increased mortality risk among foreign-born individuals observed in 2020 declined in 2021 and further decreased in 2022.^4^ Higher societal immunity (herd immunity), better individual immunity, and fewer severe COVID-19 cases might explain the lower prevalence of hospital-registered COVID-19 cases some years into the COVID-19 pandemic.^33^ Still, a gap persisted between foreign-born and Swedish-born individuals in hospital-registered COVID-19 cases. The risk, however, was much lower in 2022 than in 2020. One probable reason is that infections and vaccinations have resulted in acquired immunity to COVID-19 in the population and, ultimately, in higher mortality rates among foreign-born individuals, observed only in the first year of the pandemic.^4^

One factor to consider is that foreign-born individuals may have worse initial health status than Swedish-born individuals due to a lack of preventive programmes and inadequate healthcare in their countries of origin prior to emigration.^10^ This can make some groups of foreign-born individuals, such as non-European immigrants, more prone to being severely ill from COVID-19. However, this might be contradicted by the healthy migrant hypothesis, which suggests that foreign-born individuals who immigrate to Sweden and obtain a residence permit are healthier than those who do not reach Sweden.^34^ The low mortality rate observed after COVID-19 in this study may indicate a healthy migrant effect. In this study, we have only data on foreign-born individuals who received a residence permit. There appears to be a selective process throughout the entire immigration process, from the homeland to the issuance of a residence permit in the host country, implying that asylum seekers have somewhat less health than migrants in general.^35^ There may have been an even larger difference between foreign-born and Swedish-born individuals if also asylum-seekers had been included. In most contact occupations, foreign-born individuals had a considerably lower vaccination rate than Swedish-born individuals; this may also have contributed to the higher hospital-registered COVID-19 prevalence among foreign-born individuals in these occupations. In hindsight, foreign-born individuals should have been prioritised for preventive measures from the beginning of an epidemic.

### Strengths and limitations of this study

The major strength of our study was its population-based cohort, comprising all foreign-born adult individuals (over 1.6 million) registered as residents in Sweden as of 31 December 2019. In addition, we could include all Swedish-born adults, which would yield high statistical power given the over 6 million individuals. Furthermore, the study was based on high-quality registers from several authorities, enabling us to consider a range of reliable demographic and work-related factors.^18 21 22 36^ Another major strength of our methodology is that we can compare individuals in the same trade, presumably with the same exposure, who differ only in their country of birth. Furthermore, we followed all primary vaccinated individuals from 12 months post-immunisation, providing a more accurate assessment of COVID-19 during follow-up to 31 December 2022, as it compensates for the lag in vaccination rates among foreign-born individuals compared with Swedish-born individuals. Finally, we have only included those with confirmed COVID-19 by PCR test (ICD-10: U07.1),^20^ minimising misclassification of the outcome.

Limitations include only accessing foreign-born people living in Sweden with a residence permit. The group without a residence permit may be even more vulnerable to contracting COVID-19, but escaped our follow-up. Further studies may, therefore, also target this group. Hospital treatment in Sweden was reserved for those with the most severe COVID-19 symptoms; others were advised to stay home or use the primary healthcare until the infection had passed.^24^ Hence, in this study, we covered the most severe cases of COVID-19, including both inpatients and outpatients at hospitals in Sweden. We cannot, therefore, assess what the differences in COVID-19 rates look like in less severe cases. This may have lowered the risk estimates, as these individuals would be considered uninfected, but only if there is a difference in undetected COVID-19 cases between foreign-born and Swedish-born individuals. Therefore, in future research, those with less acute disorders may be of interest when assessing the full consequences of COVID-19 on a foreign-born population. We used the definitions ‘foreign-born’ for individuals born outside Sweden and ‘Swedish-born’ for individuals born in Sweden. This means that children of foreign-born individuals born in Sweden will be regarded as Swedish-born. If those children exhibit the same health and vaccination behaviour as their foreign-born parents, this may reduce the differences in estimates between foreign-born and Swedish-born individuals. Furthermore, analyses were restricted to working-age adults to align with the labour-market focus; this choice is appropriate for the study’s aims but results in few mortality events and limits generalisability to older age groups. Finally, data on individuals’ COVID-19 infection status before vaccination were generally unavailable. Prior COVID-19 infection can confer immune protection comparable to that induced by vaccination, thereby reducing the risk of severe COVID-19.

## Conclusions

The gap in hospital-registered COVID-19 incidence between foreign-born and Swedish-born individuals narrowed after the vaccination campaign that began at the start of 2021. Some occupations, such as personal transport workers, teaching professionals and service workers, remained at higher risk. Also, low education level and large household size increased the risk of severe COVID-19 after mutual adjustment in the working-age population. Furthermore, the increased 30-day mortality among working-age foreign-born individuals with confirmed COVID-19, as seen in the first year of the pandemic, was not seen in 2021 and 2022. The vaccine appears to have levelled out most of the differences between foreign-born and Swedish-born individuals seen in 2020.

## Supplementary material

10.1136/bmjopen-2026-118005 online supplemental file 1

## Data Availability

No data are available.
