# Supplementary material for: Did the vaccination campaign in Sweden narrow the gap in hospital-registered COVID-19 cases between foreign-born and Swedish-born individuals? A register-based cohort study of adults
Source: BMJ Open. 2026 Oct 27;16(10):e118005. doi: 10.1136/bmjopen-2026-118005 (PMC13723806; doi:10.1136/bmjopen-2026-118005)

Supplementary Figure 1. Hazard Ratios (HR) for hospital-registered COVID-19 (U07.1) for foreign-born individuals with Swedish-born individuals serving as reference, with 95% Confidence Intervals (95% CI).

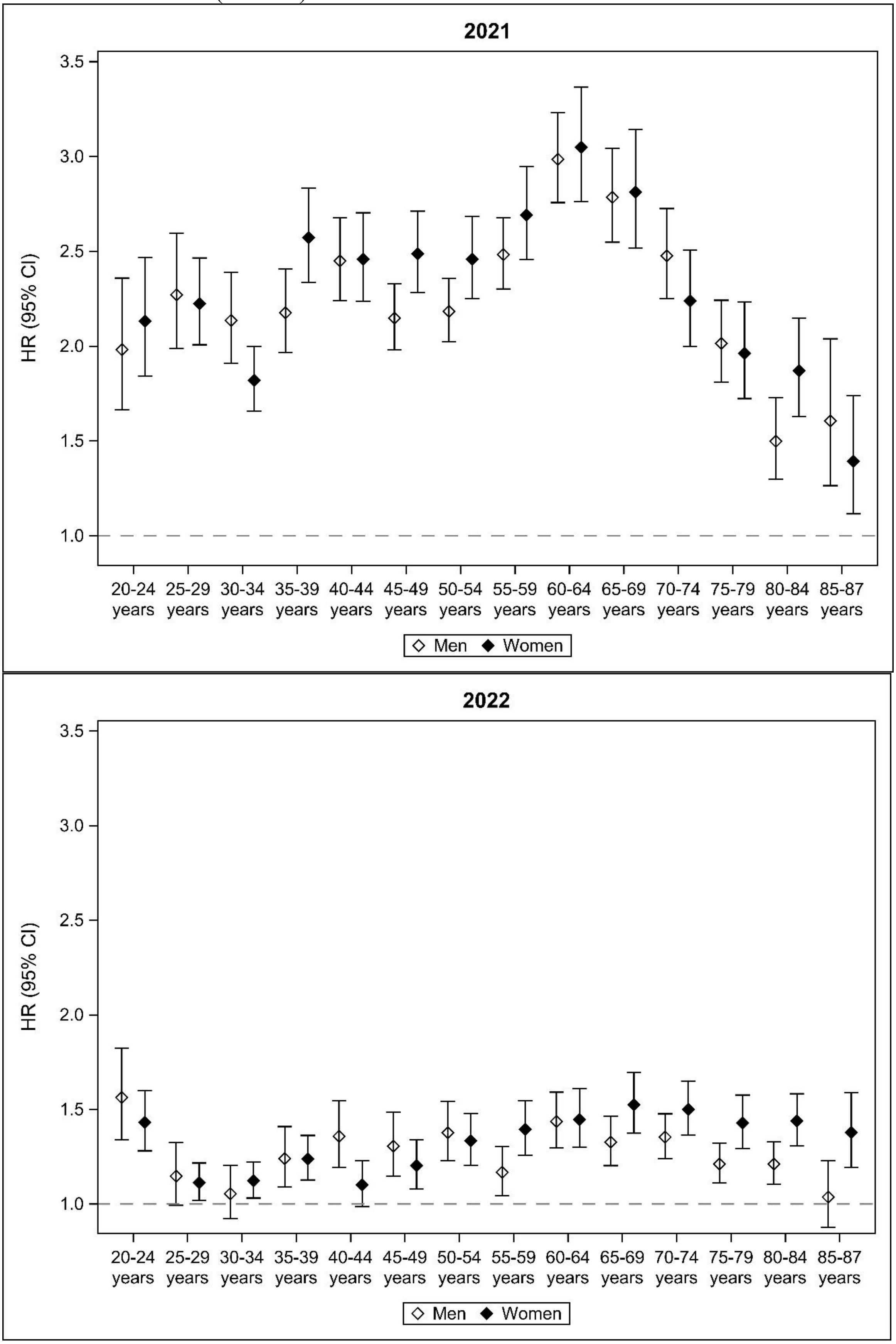

Supplementary Figure 2. Hazard Ratios (HR) for hospital-registered COVID-19 (U07.1) within 12 months after ***vaccination*** for foreign-born individuals with Swedish-born individuals serving as reference with 95% Confidence Intervals (95% CI).

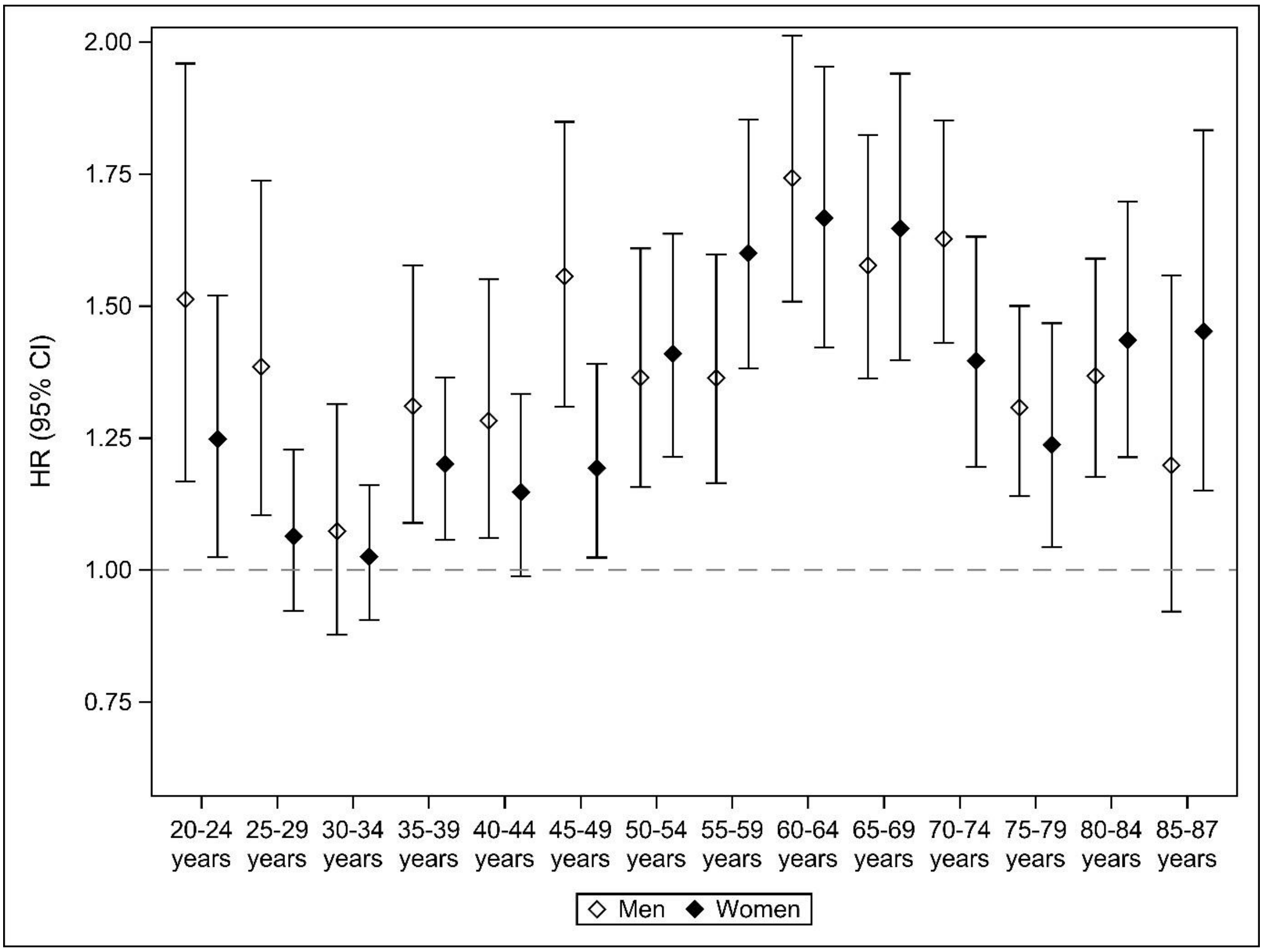

Supplementary Figure 3. Hazard Ratios (HR) for hospital-registered COVID-19 (U07.1) among unvaccinated foreign-born individuals, with Swedish-born individuals serving as reference, with 95% Confidence Intervals (95% CI).

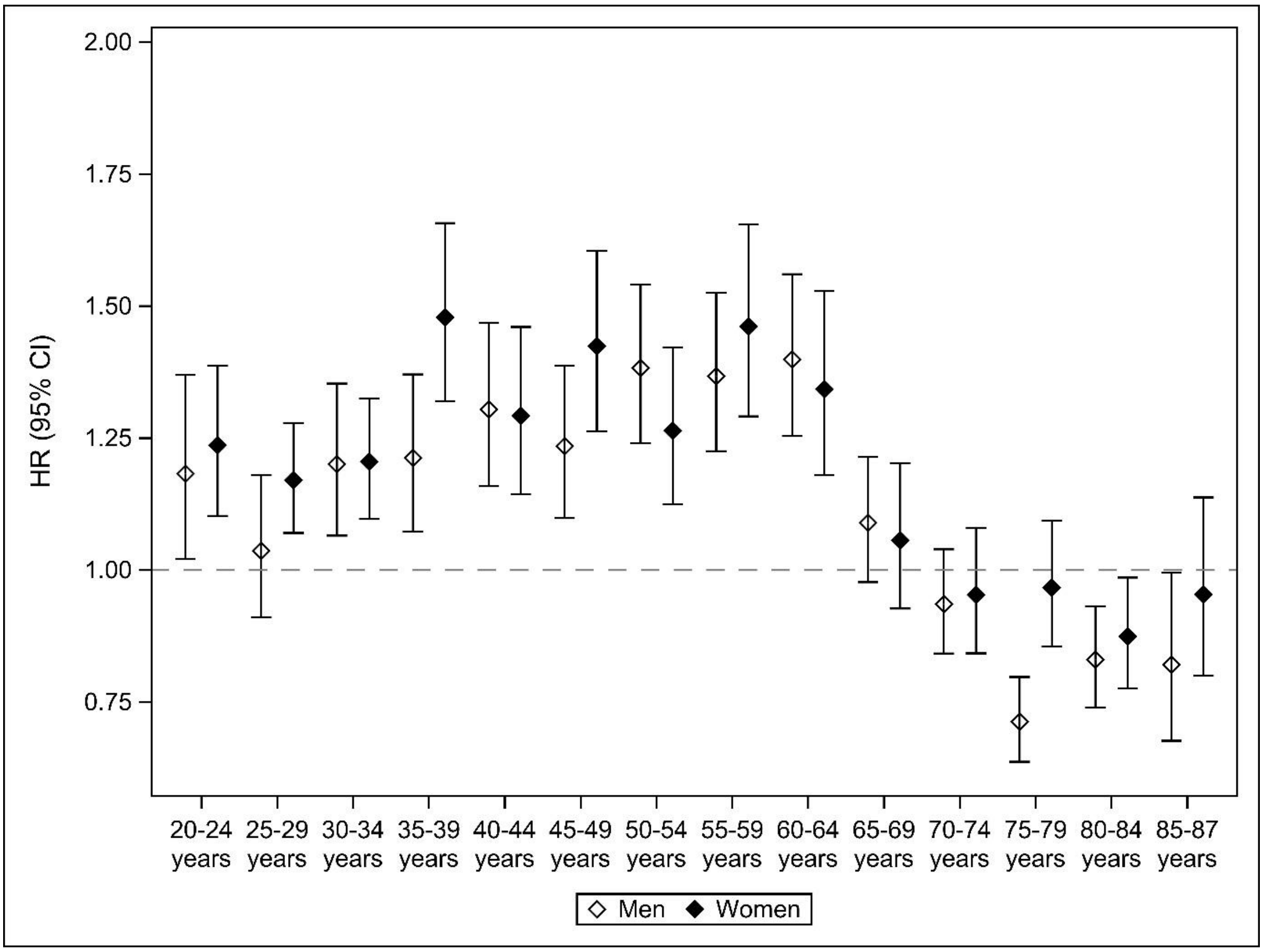

Supplement: Supplementary file 1 [file bmjopen-16-10-s001.pdf]
