# Supplementary material for: Did the vaccination campaign in Sweden narrow the gap in hospital-registered COVID-19 cases between foreign-born and Swedish-born individuals? A register-based cohort study of adults
Source: BMJ Open. 2026 Oct 27;16(10):e118005. doi: 10.1136/bmjopen-2026-118005 (PMC13723806; doi:10.1136/bmjopen-2026-118005)
Supplement: Supplementary file 2 [file bmjopen-2026-118005.reviewer_comments.pdf]

PEER REVIEW HISTORY

BMJ Open publishes all reviews undertaken for accepted manuscripts. Reviewers are asked to complete a checklist review form and are provided with free text boxes to elaborate on their assessment. These free text comments are reproduced below.

ARTICLE DETAILS

Title (Provisional)

Did the vaccination campaign in Sweden narrow the gap in hospital-registered COVID-19 cases between foreign-born and Swedish-born individuals? -a register-based cohort study of adults

Authors

Helgesson, Magnus; Tondel, Martin; Nordquist, Tobias; Svartengren, Magnus

---

VERSION 1 - REVIEW

**Reviewer**	**1**
**Name**	**Marszalek, Milena**
**Affiliation**	**Queen Mary University of London**
**Date**	**03-Jun-2026**
**COI**	**None**

This is a comprehensive study with a large fairly representative sample size. It is well structured and the results are well laid out. However, there are some areas requiring further clarification.

It would be helpful to have a list of the codesets for covid-19 diagnosis and for what defined a covid-19 vaccination. It is not explicitly stated that EHRs are used, but I would assume this is the case with the population study size. Therefore, this could be added as an appendix file.

The background should include where vaccinations are delivered for those not familiar with the swedish healthcare system. The methods briefly state that the vaccination data came from the Swedish National Vaccination register, but further information about definitions of covid-19 vaccination and codes used to define them in the EHR (as mentioned above) should be included.

The rationale for the choice of date of vaccination is not clear- the authors have chosen a week after the second vaccination. Usually, EHR coded vaccination data will give the date on which the dose is administered. Deviation from this practice should be explained. Additionally, the decision to put together partial vaccination and no vaccination should be explained- the demographic data for these groups has been combined but it would be useful

to see the numbers in two separate columns. Partial vaccination patterns are different to not receiving any vaccinations, especially in the populations that are partially vaccinated vs. not vaccinated at all.

The demographic data for occupation and household data could also be more detailed- one of the categories puts together students and no data in the missing group- technically being a student wouldn't count as a missing data field.

Household data includes number of members, but does not break households down into whether they consist of only working age adults with children, just adults or whether they include older adults too. This would help to explain why some of the HRs for covid-19 hospitalisation are lower in larger households, even though their vaccination rates are lower comparing to households with fewer members.

I also query why some co-morbidities were not included as a potential explanatory variable- i.e. T2DM, HTN, CVD. Studies looking at covid-19 mortality in the UK found strong evidence of increased covid-19 severity in people with T2DM, HTN, CVD, and some evidence of increased prevalence of these co-morbidities in some ethnic groups, especially in people of working-age. As mortality is defined as death within 30 days of hospitalisation with Covid-19 regardless of cause, it would be helpful to know about whether there is increased risk of mortality in those with HTN, CVD, T2DM. Or if this is not possible (i.e. this data is not available within the datasets), an explanation should be given.

Reviewer **2**

Name **Dzurova, Dagmar**

Affiliation **Charles University, Department of Social Geography and Regional Development**

Date **11-Jun-2026**

COI **None**

This manuscript presents an analysis of nationwide register-based data from Sweden, examining COVID-19 vaccination uptake as well as COVID-19 incidence and mortality among foreign-born and Swedish-born populations during 2021–2022. The study addresses an important and timely public health question and has the potential to contribute valuable evidence on the role of vaccination in shaping inequalities in COVID-19 outcomes across population groups. A major strength of the study is the use of comprehensive, high-quality linked national registers, which provide opportunities for population-level analyses that are rarely available in many countries.

In my view, the study is of particular interest because it draws on unique linked administrative and health data from Sweden, a country whose response to the COVID-19 pandemic attracted considerable international attention and differed in several respects

from approaches adopted elsewhere. As a result, there remains substantial scientific interest in understanding the effectiveness and equity implications of preventive measures implemented during the pandemic. For this reason, I was pleased to accept the invitation to review the manuscript, although it had already undergone peer review and the version under consideration had been revised in response to comments from two previous reviewers.

The authors have addressed many of the concerns raised during the previous review round, and the revised manuscript has improved in several respects. Nevertheless, I believe that an important limitation remains regarding the overall clarity and focus of the study. The manuscript simultaneously addresses multiple research questions—vaccination uptake, COVID-19 incidence, mortality, and differences between foreign-born and Swedish-born populations. While each of these objectives is relevant and important, their combined presentation makes the manuscript difficult to follow and, at times, obscures its central message. A clearer prioritisation of the research aims and a more focused presentation of the results would substantially improve the readability and scientific impact of the paper.

I also have two specific comments regarding the interpretation of the findings.

Discussion (pp. 18–19, “The relationship between vaccine status and hospital-registered COVID-19”): The interpretation that lower COVID-19 rates observed among some unvaccinated foreign-born individuals may be explained by infection-induced immunity acquired earlier in the pandemic appears speculative. As the authors acknowledge (p. 21, lines 14–16), information on prior SARS-CoV-2 infection before vaccination was not available in the data. Consequently, this explanation cannot be directly evaluated and should therefore be presented more cautiously as a possible interpretation rather than as a likely mechanism.

Conclusions (p. 22): Similarly, statements suggesting that the observed narrowing of inequalities may be attributable to herd immunity appear stronger than can be supported by the available evidence. Since neither previous infection history nor population immunity was directly measured, references to herd immunity should be framed as hypotheses or potential explanations rather than as conclusions emerging from the study findings.

In my conclusion, the manuscript addresses a relevant and scientifically important research question and is based on an exceptionally valuable data source. The linked national registers provide a unique opportunity to generate evidence on COVID-19 vaccination and inequalities in severe COVID-19 outcomes among migrant and native-born populations. However, I am not fully convinced that the manuscript, in its current form, fully realises the potential of these data.

My most important concern relates to the interpretation of the findings and the consistency between the evidence presented and the conclusions drawn. The principal conclusion of the paper states that “the incidence of severe COVID-19 remained higher among foreign-born individuals; however, these differences decreased as vaccination and herd immunity

expanded." While the analyses clearly demonstrate changes in inequalities over time and document differences in vaccination uptake, the role of herd immunity was not directly examined. As acknowledged by the authors, information on previous SARS-CoV-2 infections was unavailable, and no indicators of population immunity were included in the analyses. Consequently, the proposed explanation for the narrowing of inequalities cannot be empirically verified using the available data. Although herd immunity may represent a plausible hypothesis, it should not be presented as a conclusion arising from the analyses.

VERSION 1 - AUTHOR RESPONSE

Reviewer: 1

Dr. Milena Marszalek, Queen Mary University of London, Royal Free London NHS Foundation Trust

Comments to the Author:

1) This is a comprehensive study with a large fairly representative sample size. It is well structured and the results are well laid out. However, there are some areas requiring further clarification.

Author response:

We thank the reviewer for the positive assessment of our work. We appreciate the recognition of the study's comprehensive design, large population-based sample, and clear presentation of the results. We have carefully considered the points raised below and have revised the manuscript accordingly to provide further clarification where needed. We hope that these revisions have strengthened the manuscript and improved its overall clarity.

2) It would be helpful to have a list of the codesets for covid-19 diagnosis and for what defined a covid-19 vaccination. It is not explicitly stated that EHRs are used, but I would assume this is the case with the population study size. Therefore, this could be added as an appendix file.

Author response:

We thank the reviewer for this suggestion. To improve transparency, we have clarified in the Methods section how COVID-19 diagnoses and vaccination status were identified from the national registers. Because vaccination information was obtained from the National Vaccination Register, individuals were classified by recorded COVID-19 vaccine doses rather than by diagnostic or procedure codes. Relevant diagnostic codes used to identify COVID-19 outcomes have been reported in the manuscript; see also the answer to the next question. We hope that these additions improve the clarity and reproducibility of the study.

3) The background should include where vaccinations are delivered for those not familiar with the swedish healthcare system. The methods briefly state that the vaccination data came from the Swedish National Vaccination register, but further information about definitions of covid-19 vaccination and codes used to define them in the EHR (as mentioned above) should be included.

Author response:

We thank the reviewer for this helpful suggestion. We agree that additional background information on the organisation of the COVID-19 vaccination programme in Sweden will improve the manuscript's accessibility to readers unfamiliar with the Swedish healthcare system.

In the revised manuscript, we have added a subsection to the Methods section describing how COVID-19 vaccinations in Sweden were delivered through the regional healthcare systems, including primary healthcare centres, dedicated vaccination clinics, hospitals, and, when relevant, residential care facilities. Information on all administered COVID-19 vaccine doses was reported by healthcare providers to the Swedish National Vaccination Register, which is

maintained by the Public Health Agency of Sweden. The register contains information on the date of vaccination, vaccine product, dose, and the vaccinated individual's personal identity number, enabling nationwide follow-up of vaccination uptake.

We have also clarified how COVID-19 vaccination status was defined in the study and provided additional details regarding the register-based identification of vaccine doses. Furthermore, relevant diagnostic codes and outcome definitions have been added to the supplementary material to improve transparency and reproducibility.

We have inserted the following text into the manuscript (please see page 7, row 18 to page 8, row 13):

"COVID-19 vaccination programme in Sweden

During the COVID-19 pandemic, vaccinations were delivered through Sweden's publicly funded regional healthcare system, including primary healthcare centres, dedicated vaccination clinics, hospitals, and residential care settings. Every vaccine shot was provided at no cost to all citizens, with priority given to vulnerable groups such as elderly persons and individuals with known risk factors for having severe COVID-19. From 1 January 2021, reporting of all COVID-19 vaccinations to the National Vaccination Register maintained by the Public Health Agency of Sweden became mandatory under the Act on Registers of National Vaccination Programmes, Swedish Code of Statutes (SFS) 2012:453, as amended by SFS 2020:1194.[15] The register contains individual-level information, including personal identity number, vaccination date, and vaccine type, enabling nationwide monitoring of vaccination uptake and coverage.[3] Primary vaccination was defined in this study as two doses of a vaccine, regardless of manufacturer (i.e., one basic shot and one booster shot), administered within 6 weeks of the basic shot.[16 17] Because full protective efficacy after

COVID-19 vaccination is not achieved immediately, the immune system needs time to develop an adequate response. Among the two most widely used vaccines in Sweden, full protection occurred 7 days after the second dose with the Pfizer-BioNTech vaccine[16] and 14 days after the second dose with the Moderna mRNA vaccine[17]. Therefore, the shortest time to full protection, 7 days after administration of the second dose, was chosen as the primary vaccination date."

4) The rationale for the choice of date of vaccination is not clear- the authors have chosen a week after the second vaccination. Usually, EHR coded vaccination data will give the date on which the dose is administered. Deviation from this practice should be explained. Additionally, the decision to put together partial vaccination and no vaccination should be explained- the demographic data for these groups has been combined but it would be useful to see the numbers in two separate columns. Partial vaccination patterns are different to not receiving any vaccinations, especially in the populations that are partially vaccinated vs. not vaccinated at all.

Author response:

We thank the reviewer for these important comments and agree that the rationale for our classification of vaccination status should be explained more clearly.

Regarding the definition of full vaccination, we did not use the date of administration of the second vaccine dose as the date of full vaccination. Instead, individuals were considered fully vaccinated one week after receipt of the second dose, reflecting the time required for the immune response to develop and stabilise following vaccination. Our intention was to capture the period during which the vaccine was expected to provide its full protective effect, rather than the date of administration. We have clarified this rationale in the Methods section, and

also included some references (Polack et al. Safety and Efficacy of the BNT162b2 mRNA Covid-19 Vaccine. N Engl J Med. 2020;383(27):2603–15 and Baden et al. Efficacy and Safety of the mRNA-1273 SARS-CoV-2 Vaccine. N Engl J Med. 2021;384(5):403–16.

Regarding the grouping of partially vaccinated and unvaccinated individuals, we acknowledge the reviewer's point that these groups may, to some point, differ demographically and in vaccination behaviour. Our decision was based on the primary objective of comparing individuals who had achieved full vaccination status with those who had not yet reached it. However, we agree that the distinction between partial vaccination and no vaccination is informative. To improve transparency and readability, we have revised the manuscript to better explain this analytical choice (please see page 7, row 18 to page 8, row 13):

"COVID-19 vaccination programme in Sweden

During the COVID-19 pandemic, vaccinations were delivered through Sweden's publicly funded regional healthcare system, including primary healthcare centres, dedicated vaccination clinics, hospitals, and residential care settings. Every vaccine shot was provided at no cost to all citizens, with priority given to vulnerable groups such as elderly persons and individuals with known risk factors for having severe COVID-19. From 1 January 2021, reporting of all COVID-19 vaccinations to the National Vaccination Register maintained by the Public Health Agency of Sweden became mandatory under the Act on Registers of National Vaccination Programmes, Swedish Code of Statutes (SFS) 2012:453, as amended by SFS 2020:1194.[15] The register contains individual-level information, including personal identity number, vaccination date, and vaccine type, enabling nationwide monitoring of vaccination uptake and coverage.[3] Primary vaccination was defined in this study as two doses of a vaccine, regardless of manufacturer (i.e., one basic shot and one booster shot),

administered within 6 weeks of the basic shot.[16 17] Because full protective efficacy after COVID-19 vaccination is not achieved immediately, the immune system needs time to develop an adequate response. Among the two most widely used vaccines in Sweden, full protection occurred 7 days after the second dose with the Pfizer-BioNTech vaccine[16] and 14 days after the second dose with the Moderna mRNA vaccine[17]. Therefore, the shortest time to full protection, 7 days after administration of the second dose, was chosen as the primary vaccination date."

We hope these additions clarify both the rationale for the definition of the vaccination date and the handling of vaccination status categories in the revised manuscript.

5) The demographic data for occupation and household data could also be more detailed- one of the categories puts together students and no data in the missing group- technically being a student wouldn't count as a missing data field. Household data includes number of members, but does not break households down into whether they consist of only working age adults with children, just adults or whether they include older adults too. This would help to explain why some of the HRs for covid-19 hospitalisation are lower in larger households, even though their vaccination rates are lower comparing to households with fewer members.

Author response:

We thank the reviewer for this valuable comment and agree that additional clarification regarding the occupation and household variables is warranted.

Regarding the occupation variable, we would like to clarify that the "missing" category does not solely represent missing data in the conventional sense. In the register data, relevant occupation information is unavailable for individuals not in the workforce, such as students and the unemployed. As a result, these individuals are included in the same category because

no occupational classification can be assigned to them. Since occupation is a key variable in the present study, we chose to retain this category rather than exclude these individuals from the analyses. We acknowledge that the current terminology may be confusing and have revised the manuscript and tables to clarify the composition of this category and the underlying reasons for its use. Please see page 9, row 24 to page 10, row 1, for a clarification statement of this in the methods section.

Regarding household composition, we agree that more detailed information on household structure could have provided additional insights into the observed associations. However, the available data allowed us to classify households only by the number of household members, and information on household composition (e.g., households consisting of adults only, families with children, or multigenerational households including older adults) was not available in a form suitable for the present analyses. We have added a clarification of this limitation to the revised manuscript. Please see page 9, rows 19-20:

"From the register, we did not retrieve the age for each household member, only household size in 2019."

We hope that these revisions provide a clearer understanding of how the occupation and household variables were constructed and interpreted, and thereby facilitate the interpretation of the reported hazard ratios and vaccination patterns.

6) I also query why some co-morbidities were not included as a potential explanatory variable- i.e. T2DM, HTN, CVD. Studies looking at covid-19 mortality in the UK found strong evidence of increased covid-19 severity in people with T2DM, HTN, CVD, and some evidence of increased prevalence of these co-morbidities in some ethnic groups, especially in people of working-age. As mortality is defined as death within 30 days of hospitalisation with

Covid-19 regardless of cause, it would be helpful to know about whether there is increased risk of mortality in those with HTN, CVD, T2DM. Or if this is not possible (i.e. this data is not available within the datasets), an explanation should be given.

Author response:

We thank the reviewer for this important comment. We agree that underlying conditions such as type 2 diabetes mellitus (T2DM), hypertension (HTN), and cardiovascular disease (CVD) are established risk factors for severe COVID-19 outcomes and may contribute to differences in mortality risks between population groups.

We would like to clarify that, in line with the Swedish national monitoring and reporting practices during the pandemic, COVID-19 mortality was defined as death occurring within 30 days of a confirmed COVID-19 diagnosis. This definition has been widely used in Swedish register-based analyses to enable consistent surveillance and comparison over time. While this approach does not establish COVID-19 as the direct cause of death in every case, it has been the standard outcome definition used in national reporting.

We first examined whether the prevalence of cardiovascular diseases (ICD-10 chapter I) and respiratory diseases (ICD-10 chapter J) differed between foreign-born and Swedish-born individuals. We found no major differences between the groups; if anything, Swedish-born individuals had a slightly higher prevalence of diagnoses in these disease categories. We have clarified this in the revised manuscript. Please see page 8, row 23 to page 9, row 1:

"Because the prevalence of cardiovascular or pulmonary diseases was similar among both foreign- and Swedish-born individuals, we did not include additional disorders in the analyses."

We hope this clarifies the definition of COVID-19 mortality and the scope of the analyses for the reader.

Reviewer: 2

Dr. Dagmar Dzurova, Charles University

Comments to the Author:

1) This manuscript presents an analysis of nationwide register-based data from Sweden, examining COVID-19 vaccination uptake as well as COVID-19 incidence and mortality among foreign-born and Swedish-born populations during 2021–2022. The study addresses an important and timely public health question and has the potential to contribute valuable evidence on the role of vaccination in shaping inequalities in COVID-19 outcomes across population groups. A major strength of the study is the use of comprehensive, high-quality linked national registers, which provide opportunities for population-level analyses that are rarely available in many countries. In my view, the study is of particular interest because it draws on unique linked administrative and health data from Sweden, a country whose response to the COVID-19 pandemic attracted considerable international attention and differed in several respects from approaches adopted elsewhere. As a result, there remains substantial scientific interest in understanding the effectiveness and equity implications of preventive measures implemented during the pandemic. For this reason, I was pleased to accept the invitation to review the manuscript, although it had already undergone peer review and the version under consideration had been revised in response to comments from two previous reviewers.

Author response:

We thank the reviewer for their careful and thoughtful assessment of our manuscript. We greatly appreciate the positive evaluation of the study's relevance, originality, and potential contribution to the literature on COVID-19-related health inequalities. We are particularly encouraged that the reviewer recognises the value of the nationwide linked register data and the unique opportunity these data provide to examine vaccination uptake and COVID-19 outcomes at the population level.

We also appreciate the reviewer's observation regarding the international relevance of the Swedish context. As noted, Sweden's pandemic response attracted considerable attention, and we agree that there remains an important need for evidence on both the effectiveness and equity implications of preventive measures implemented during this period.

2) The authors have addressed many of the concerns raised during the previous review round, and the revised manuscript has improved in several respects. Nevertheless, I believe that an important limitation remains regarding the overall clarity and focus of the study. The manuscript simultaneously addresses multiple research questions—vaccination uptake, COVID-19 incidence, mortality, and differences between foreign-born and Swedish-born populations. While each of these objectives is relevant and important, their combined presentation makes the manuscript difficult to follow and, at times, obscures its central message. A clearer prioritisation of the research aims and a more focused presentation of the results would substantially improve the readability and scientific impact of the paper.

Author response:

We thank the reviewer for this thoughtful comment. We acknowledge that the manuscript addresses several related outcomes, including vaccination uptake, COVID-19 incidence, mortality, and differences between foreign-born and Swedish-born populations. However,

these aspects were intentionally included because they are closely interconnected and together provide a more comprehensive understanding of the research question. Studying these outcomes separately would risk overlooking important relationships between vaccination patterns and subsequent health outcomes across population groups. Another rationale for broad inclusion is that the current study is a follow-up to an article published in OEM in 2024 on the same topic. The previous study presented the situation before the vaccination campaign against COVID-19 had started (Tondel et. al: COVID-19: incidence and mortality in Sweden comparing all foreign-born to all Swedish-born individuals in different occupations in an unvaccinated cohort of year 2020. OEM. 2024;81(3).)

To address the reviewer's concern, we have revised the manuscript to more clearly emphasise this overarching perspective. Specifically, we have clarified in the Aim section that the study is designed to investigate these interrelated dimensions within a unified analytical framework, please see page 6, rows 15-24:

"Therefore, the aims were to study differences between foreign-born and Swedish-born individuals regarding the following interrelated research questions: 1) risk factors and differences between foreign-born and Swedish-born individuals regarding hospital-registered COVID-19, 2) vaccine rates in 2021 for COVID-19 in foreign-born and Swedish-born individuals, 3) the relationship between vaccine status in 2021 and subsequent one-year hospital-registered COVID-19 in foreign-born and Swedish-born individuals and 4) differences between vaccinated and unvaccinated foreign-born and Swedish-born individuals in mortality within 30 days of a hospital-registered COVID-19. In all the above relationships, the roles of sex, age, educational level, household size, and occupation should be considered."

We hope that these revisions make the rationale for including multiple outcomes more transparent and improve the manuscript's overall readability.

3) I also have two specific comments regarding the interpretation of the findings.

Discussion (pp. 18–19, "The relationship between vaccine status and hospital-registered COVID-19"): The interpretation that lower COVID-19 rates observed among some unvaccinated foreign-born individuals may be explained by infection-induced immunity acquired earlier in the pandemic appears speculative. As the authors acknowledge (p. 21, lines 14–16), information on prior SARS-CoV-2 infection before vaccination was not available in the data. Consequently, this explanation cannot be directly evaluated and should therefore be presented more cautiously as a possible interpretation rather than as a likely mechanism.

Author response:

We thank the reviewer for this important observation. We agree that, given the lack of information on prior SARS-CoV-2 infection in our dataset, we cannot directly assess whether infection-induced immunity contributed to the lower hospital-registered COVID-19 rates observed among some unvaccinated foreign-born groups.

Our intention was to suggest this as one possible explanation for the observed pattern rather than to present it as a confirmed or likely mechanism. To address this concern, we have revised the discussion to use more cautious wording and explicitly frame infection-induced immunity as a hypothesis that may warrant further investigation. We also emphasise that alternative explanations may exist and that our data do not allow us to determine the relative contribution of prior infection to the observed associations.

These revisions have been made to ensure the interpretation remains consistent with the available data and the study's limitations.

4) Conclusions (p. 22): Similarly, statements suggesting that the observed narrowing of inequalities may be attributable to herd immunity appear stronger than can be supported by the available evidence. Since neither previous infection history nor population immunity was directly measured, references to herd immunity should be framed as hypotheses or potential explanations rather than as conclusions emerging from the study findings.

Author response:

We appreciate the reviewer's thoughtful comment and agree that this is an important consideration. We agree that, given the lack of information on prior SARS-CoV-2 infection in our dataset, we cannot directly assess whether infection-induced immunity contributed to the lower hospital-registered COVID-19 rates observed among some unvaccinated foreign-born groups. Our intention was to suggest this as one possible explanation for the observed pattern rather than to present it as a confirmed or likely mechanism. To address this concern, we have revised the conclusion.

5) In my conclusion, the manuscript addresses a relevant and scientifically important research question and is based on an exceptionally valuable data source. The linked national registers provide a unique opportunity to generate evidence on COVID-19 vaccination and inequalities in severe COVID-19 outcomes among migrant and native-born populations. However, I am not fully convinced that the manuscript, in its current form, fully realises the potential of these data.

Author response:

We thank the reviewer for this overall assessment and for recognising the value and limitations of the national register data used in this study. We appreciate the concern that the

manuscript may not have fully communicated the potential of these data in its previous version.

The study design was deliberately chosen to provide a comprehensive assessment of inequalities in vaccination uptake, hospital-registered COVID-19, and COVID-19-related mortality among foreign-born and Swedish-born populations. We consider these outcomes closely interconnected and believe that analysing them within a common framework offers important insights that would be less apparent if each were studied separately.

In response to comments from both the current and previous review rounds, we have further revised the manuscript to make the rationale, aims, and analytical approach more explicit. In particular, we have clarified in the Aim and Methods sections how the different study components contribute to the overarching research question. We hope these revisions make the study's focus clearer and better highlight the strengths and opportunities of the linked national register data.

We are grateful for the reviewer's comments, which have helped us improve the manuscript's clarity and overall presentation.

7) My most important concern relates to the interpretation of the findings and the consistency between the evidence presented and the conclusions drawn. The principal conclusion of the paper states that "the incidence of severe COVID-19 remained higher among foreign-born individuals; however, these differences decreased as vaccination and herd immunity expanded." While the analyses clearly demonstrate changes in inequalities over time and document differences in vaccination uptake, the role of herd immunity was not directly examined. As acknowledged by the authors, information on previous SARS-CoV-2 infections was unavailable, and no indicators of population immunity were included in the analyses.

Consequently, the proposed explanation for the narrowing of inequalities cannot be empirically verified using the available data. Although herd immunity may represent a plausible hypothesis, it should not be presented as a conclusion arising from the analyses.

Author response:

We thank the reviewer for all insightful suggestions to improve the manuscript and agree that the previous wording may have implied a stronger conclusion than the available data support. As the reviewer correctly points out, we did not have access to information on prior SARS-CoV-2 infections and therefore could not directly assess herd immunity or population-level immunity in our analyses.

Our intention was to propose expanding vaccination and immunity acquired through prior infection as a possible explanation for the observed reduction in inequalities over time, rather than to present herd immunity as a finding directly demonstrated by our results. We acknowledge that this distinction was not sufficiently clear in the previous version of the manuscript.

In response to this comment, we have revised both the Discussion and Conclusion sections to present herd immunity more cautiously as a hypothesis that may contribute to the observed patterns, rather than as a conclusion arising from the analyses.

More broadly, following comments from both the current and previous review rounds, we have further clarified the study design, aims, and interpretation of the findings throughout the manuscript. We hope that these revisions provide a clearer distinction between findings directly supported by the analyses and potential explanations put forward to aid interpretation, thereby strengthening the consistency between the evidence presented and the con

---

VERSION 2 - REVIEW

Reviewer 1

Name Marszalek, Milena

Affiliation Queen Mary University of London

Date 25-Sep-2026

COI

Thank you for addressing my queries in this revision of the paper. I have no further queries to add.
